# What Are the Factors Associated with Nonadherence to Medications in Patients with Chronic Diseases?

**DOI:** 10.3390/healthcare9091237

**Published:** 2021-09-20

**Authors:** Abdel Qader Al Bawab, Walid Al-Qerem, Osama Abusara, Nimer Alkhatib, Maha Mansour, Robert Horne

**Affiliations:** 1 Faculty of Pharmacy, Al-Zaytoonah University of Jordan, Amman 11733, Jordan; waleed.qirim@zuj.edu.jo (W.A.-Q.); o.abusara@zuj.edu.jo (O.A.); n.alkhatib@zuj.edu.jo (N.A.); mahamosho@yahoo.com (M.M.); 2 Centre for Behavioral Medicine, UCL School of Pharmacy, University College London, London WC1H 9JP, UK; r.horne@ucl.ac.uk

**Keywords:** adherence, beliefs, medications, regression

## Abstract

**Introduction**: Adherence to medications is very crucial for an optimized clinical outcome in the management of chronic diseases. Beliefs about medications and other factors can significantly affect adherence to chronic medications. The objective of the present research was to identify the associated factors of adherence to medication in Jordanian patients with chronic diseases utilizing a stepwise binary logistical regression model. **Methods**: A cross-sectional study was carried out between November 2018 and March 2020. The participants were reached from secondary and tertiary care setting clinics in Jordan. The recruited patients were asked to report their attitudes of adherence to medications and beliefs about medications via filling out the MARS-5 and BMQ-specific tools. Sociodemographic data were also collected from the recruited patients and included in the regression model. A stepwise binary logistical regression model was applied to identify the associated factors of adherence to chronic medications in the tested sample. **Results**: A total of 485 patients who met the inclusion criteria were recruited. The mean age of the participants was 57.14 (age ranged from 22 to 82 years). Around 39% of the participants were older than 65 years. Most of the patients were either hypertensive or diabetic (35.7% and 32.2%, respectively). The logistic regression model indicated that necessity beliefs are strongly associated with adherence (OR 4.22), while concerns beliefs, dosage frequency and having medical insurance were negatively associated with adherence (OR 0.73, 0.74 and 0.26, respectively), with a *p*-value ≤ 0.05. **Conclusions**: Both the MARS-5 and BMQ-specific questionnaires were applied successfully on the tested sample. Better attention should be paid to the logistic regression model variables that were associated with adherence in order to guarantee optimal treatment outcomes in the treatment of chronic diseases.

## 1. Introduction

Chronic diseases are prevalent in a majority of the elderly population [1]. These patients are involved in “polypharmacy”, as they receive multiple medications for the treatment of various chronic diseases [2]. Polypharmacy would influence elderly patients’ attitudes towards medications, hence affecting adherence to medications [3]. Nonadherence to therapeutic regimens recommended by healthcare providers is a major health problem, since adherence to these regimens is essential for the success of treatment plans and achieving the desired clinical outcomes [4].

Generally, the adherence rate to treatments in patients with acute conditions is much greater compared to adherence in patients with chronic diseases. The adherence rate is reported to fall markedly after six months from initiating a treatment regimen [5]. Knowing that adherence to medical recommendations will ultimately lead to treatment successes, it is imperative to address nonadherence behaviors by patients, as it increases the risk of treatment failure, suboptimal treatment outcomes, treatment and readmission costs and even the patients’ lives [6].

There are various determinants that influence adherence to medications. Patient, disease and medication-related factors may impact adherence and, hence, result in substandard adherence rates among adults with chronic diseases, as studies have reported [7]. Moreover, the success of a therapeutic regimen is achieved only when patients adhere precisely to them. However, as patients develop their own views regarding the use of medications, they tend to make their particular decisions based on their beliefs and practices [8]. This will eventually compromise their adherence to medications. 

In 2018, noncommunicable diseases were the cause of 78% of deaths in Jordan [9]. In 2015, the top chronic diseases related to death in Jordan were: ischemic heart disease, cerebrovascular disease, diabetes, congenital defects and chronic kidney disease [10]. As in most countries around the world, the issue of the lack of adherence towards medications in chronically ill patients is problematic in Jordan.

There is a need to measure the attitude or beliefs toward medications and whether these beliefs are positive or negative and how, in the long term, this is affected by the number or type of chronic diseases. These can be addressed partly using “Beliefs about Medications Questionnaire (BMQ)”, a tool used to measure the attitude/beliefs in a narrative estimate way [11]. BMQ can assess the patient’s attitude toward a particular medication and their attitude toward medications in general. Furthermore, BMQ can assess positive beliefs as measured by essential feelings and measure negative beliefs by measuring fears from medications. This can be achieved using a simple structure that can be utilized in any setting and is constructed to improve patient’s practices and, consequently, optimize the desired therapeutic results of a treatment plan [12,13].

Adherence to medications can be assessed using many approaches. Methods to measure adherence include therapeutic drug monitoring (TDM), electronic counting devices, pick-up/refill rates and self-report questionnaires. Administering questionnaires to assess adherence is considered one of the easiest methods despite the fact that administered questionnaires may overestimate adherence due to “social desirability” and the “white coat effect”. Nonetheless, the Medication Adherence Report Scale (MARS-5) is a well-validated tool that has been extensively used in different clinical settings [14].

Herein, the objective of this study is to explore the effect of the recruited patients’ characteristics and their health beliefs on medication adherence. Basically, we are studying the adherence to chronic medications in differently aged adult patients, addressing beliefs about medications and investigating the association between beliefs about medications and other patients’ characteristics (sociodemographic) to the extent of adherence. This study supports and guides the upgrading of future healthcare interventions to improve health outcomes for patients suffering from chronic diseases in Jordan.

## 2. Materials and Methods

### 2.1. Study Design and Participants

This is an observational cross-sectional study that adhered to the STrengthening the Reporting of OBservational studies in Epidemiology (STROBE) guidelines. It involved 485 recruited patients who were diagnosed with one or more chronic diseases and took prescribed medications for at least one year (as per the CDC definition) and were aged 20 years or older. Participants with chronic diseases were recruited from secondary and tertiary care settings clinics across Jordan.

The healthcare system in Jordan consists of 2 main sectors: the public sector and the private sector. Both sectors comprise hospitals, primary care clinics, pharmacies and other auxiliary services. Primary healthcare clinics in Jordan provide quick access to medical care that offers a wide array of medical services, including vaccination, maternity and childcare and chronic disease management services. Yet, a weighty share of healthcare in Jordan is provided through programs led by the United Nations, such as The United Nations Relief and Works Agency (UNRWA). Around 55% of the overall population are insured. The majority of Jordanians have public sector insurance. The remainder of the population is either noninsured, have coverage via private insurance or other sources like UNRWA. All children less than 6 years old and adults aged 60 years or older are automatically insured with the public health care sector [15].

The clinics involved in the present research include specialties like cardiovascular, respiratory, internal medicine and endocrinology. Chronic disease diagnoses were confirmed from patient files. Patients who were severely ill and could respond and patients who were less than 20 years old were not included in the study.

The setting of the current study was compliant with the ethical standards of the Declaration of Helsinki guidelines that were stated by the World Medical Association. The study protocol was approved by the Institutional Review Board (IRB) of Al-Zaytoonah University of Jordan (approval reference number: 07.02.2019). A signed consent form was obtained from the participant who agreed to take part in this study. Every participant provided a signed consent form after reading the study information sheet. The confidentiality of all the participants was well-maintained throughout the study.

### 2.2. Data Collection

The first part of the questionnaire consisted of queries to collect sociodemographic data about the participants. These included: age, gender, income level, educational level, diagnosis, duration of illness, the number of medications, type of medications and insurance status. Medication prices were estimated as per local prices in Jordanian Dinar (JOD).

A self-reported questionnaire (MARS-5) was included in the second part of the questionnaire and used to assess the participants’ levels of adherence toward their prescribed medications. The MARS-5 has been widely used in studies on a variety of chronic illnesses, including type two diabetes, hypertension and chronic obstructive pulmonary disease [14]. The MARS-5 is composed of five questions about “forgetting”, “changing of dosages”, “stopping”, “skipping” and “using medication less than what is prescribed”. The study subjects indicated the frequency as “always”, “often”, “sometimes”, “rarely” or “never” for each question, with ascending scores from “always” (1 point) to “never” (5 points). The scores for each of the five questions were aggregated to give the final score, which ranged from 5 to 25 points. The cutoff point of adherence in the current work was determined to be ≥80% of the aggregated MARS-5 scores for the studied population (i.e., MARS-5 score ≥ 20).

The third part included a self-reported questionnaire; the Beliefs about Medicines Questionnaire (BMQ-specific) [16]. This well-validated tool is used to measure patients’ perceptions and insights about a particular medication in more definite situations, such as chronic illnesses. BMQ-specific consists of 11 items that incorporate two subscales: the Specific-Necessity subscale that assesses the patient’s beliefs about the necessity and need of the prescribed medication and the Specific-Concerns subscale, which addresses the patients’ concerns and worries regarding the potential adverse outcomes from the medications’ use. The respondents indicated their degree of agreement with each statement about medicines on a five-point Likert scale, in which score 1 represented a strong disagreement and 5 epitomized a strong agreement. The scores obtained for the individual items within each scale were calculated as the mean to give a scale score in which the higher scores indicated stronger beliefs in the concepts represented by the scale. BMQ was developed by Professor Robert Horne and his colleagues as a method for assessing cognitive representations of medication. It has been confirmed that high concern and low necessity scores are correlated with high levels of nonadherence in a number of chronic illnesses.

Both the MARS-5 and BMQ-specific questionnaires were translated from the English language to the Arabic language, and the translations were validated by the back-translations technique and involved three qualified independent translators. The internal consistency of the questionnaires was confirmed by calculating the Cronbach’s alpha (0.89 for factor 1 (Necessity) and 0.93 for factor 2 (concerns) of the BMQ and 0.89 for the MARS-5 questionnaire, consequently). Furthermore, deleting any item of any of the questionnaires did not improve their reliability.

A well-trained research assistant (pharmacist) was employed to administer the questionnaires to the recruited patients who met the inclusion criteria in order to extract their own perceptions in a neutral manner. The inclusion criteria to participate in the present study were: patients who were 20 years or older with at least one chronic disease who were taking at least one chronic medication and who completely filled out the administered questionnaires.

### 2.3. Data Analysis

The analysis was performed using IBM SPSS Statistics software version 21 (Armonk, NY, USA). Only complete datasets were included in the statistical analysis. Continuous variables were tested for the normality of distribution before the analysis. The data were presented as the frequency (%) and mean ± standard deviation.

A stepwise binary logistical regression model was used to identify the associated variables of adherence to chronic medications, where the adherence level (adherent vs. nonadherent) was assigned as the independent variable. The dependent variables included in the model were: gender, educational level, income level, medical insurance, necessity score, concerns score, frequency of taking medications, age and disease duration. The multicollinearity of the final model variables was evaluated by computing the Variance Inflation Factor (VIF). The VIF values produced were all below 10, which implied the absence of a multicollinearity between the model variables [17]. Conditional forward stepwise regression was applied to identify the significantly associated variables. No variables were forced into the regression model. A univariate regression analysis for each of the insignificant variables confirmed the finding that these variables irrelevantly associated with the model’s independent variables.

An attitudinal analysis of the beliefs about medications was also conducted. The individual’s scores of the two BMQ domains were dichotomized at their midpoint into “high” and “low” groups, then combined to form four attitudinal groups: accepting (showing high necessity and low concern scores), ambivalent (showing high necessity and high concern scores), indifferent (showing low necessity and low concern scores) and skeptical (showing low necessity and high concern scores).

### 2.4. Sample Size Calculations

The following equation was used to compute the minimum sample size required to conduct ordinal regression: 50 + 8P, where P is the number of associated factors. The original aim of the study was to evaluate the association of the eighteen variables with the adherence level (Table 1). Therefore, the minimum required sample size to reliably conduct the statistical analysis of the studied sample was 194 [18].

## 3. Results

### 3.1. Demographic Characteristics of the Participants

During the study period, a total of 485 patients who met the inclusion criteria and agreed to sign the consent form after reading the study information sheet were recruited. The mean age of the participants was 57.14 (±12.81) and ranged between 22 and 82 years. More than one-third of the participants (38.9%) were chronologically elderly (i.e., >65 years old). Gender was distributed equally among the participants (50.7% were male).

The majority of participants (77.1%) had intermediate monthly incomes according to the Jordanian Department of Statistics, and 40% of participants had completed a bachelor’s degree program. Furthermore, 22% of the participants had two or more medical conditions. Hypertension was the most common disease among the participants (35.7%), followed by diabetes (32.2%). The number of patients with polypharmacy (defined as taking two or more medications per day [19]) was 363 (74.86%). The participant demographic characteristics are summarized in Table 1.

### 3.2. MARS-5 and BMQ-Reported Responses Portrayals

The reported answers to MARS-5 are presented in Table 2. Some (37.1%) of the participants reported that they sometimes forgot to take their medications, and about 20% of the participants reported that they always or often take less medication than prescribed. The statement with the highest mean was “I decide to skip a dose”, while the statement with the lowest mean was “I forgot to take them”. Any patient with a score below 20 was considered nonadherent, while a patient a score 20 or higher was considered adherent in this study. Accordingly, the adherence rate was calculated to be 32% of the studied sample.

Participants’ responses to the BMQ items are shown in Table 3. The item with the highest mean for a specific necessity was for “My health, at present, depends on my medicine” (4.05 ± 0.94), while the lowest was for “My health in the future will depend on my medicine” (3.33 ± 0.76). For specific concerns items, the highest mean was for “This medicine gives me unfavorable side effects” (3.59 ± 0.97), and the lowest was for “Having to take medicine worries me” (3.02 ± 0.90).

### 3.3. Logistic Regression Model

The results of the logistic regression of the adherence level are shown in Table 4. The increasing necessity mean will significantly increase the odds of the patient to be adherent (odds ratio 4.22 (CI = 2.59–6.87, *p* < 0.01)). On the other hand, an increasing concerns mean will significantly decrease the odds of the adherence level (odds ratio 0.73 (CI = 0.56–0.95, *p* = 0.02)). Similarly, increasing the frequency of medication taking will significantly decrease the adherence level (odds ratio 0.74 (CI = 0.65–0.84, *p* < 0.01)). Patients with medical insurance had significantly lower odds of adherence compared to those with no insurance (odds ratio 0.26 (CI = 0.16–0.4, *p* < 0.01)). No significant interaction was found between the significant variables included in the final regression model.

**Figure 1:**
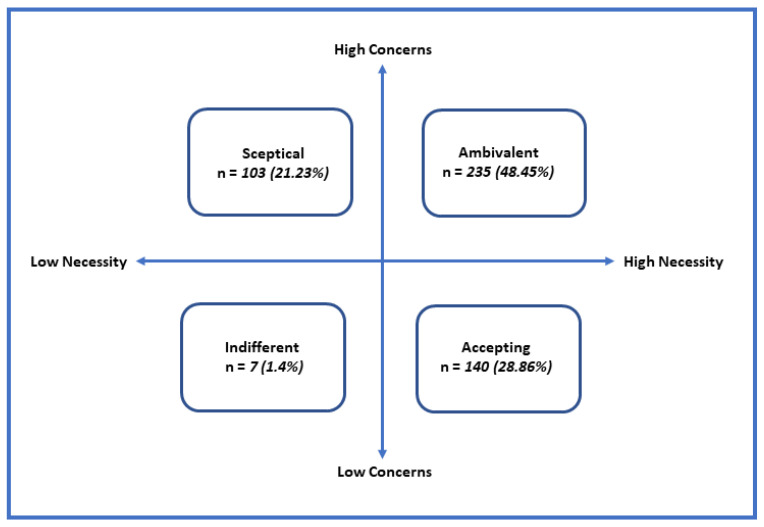
Attitudinal analysis of BMQ-specific findings in the studied sample.

An attitudinal analysis of the BMQ scores (Figure 1) revealed interesting information about how patients think about their medications. Around half of the patients were ambivalent toward their medications. They expressed mixed feelings and, to some extent, contradictory ideas in terms of the necessities and concerns about their medications. Around one-third of the patients expressed an adequate acceptance toward the usage of their medications. Around one-fifth of the patients were not convinced and had doubts about the usage of their medications, while less than 2% were offhand about their medications.

## 4. Discussion

The main objective of the present research was to identify the significant associated factors of adherence to medications in patients with chronic diseases utilizing a stepwise binary logistical regression model. BMQ-specific necessity and BMQ-specific concerns were incorporated in a regression model and related to the level of reported adherence. Both the necessity and concern scores of BMQ-specific were able to differentiate adherent and nonadherent patients with chronic diseases who were identified using the MARS-5 adherence to medication questionnaire.

The determinants of each BMQ factor implied that patients’ ideas about medication were coherent in “common sense” terms. For example, the patients were more likely to have strong beliefs about the necessity of their medication if it was perceived to affect their symptoms. In the current study, the patients had strong positive attitudes and beliefs that medications are important for good health (specific-necessity score 3.67) but also reported fears of consequences related to taking medications regularly, like side effects, duration of the use and dependence to medications (specific-concerns score 3.28). The BMQ-specific item with the highest necessity was “my health, at present, depends on my medicine,” while the item “my health in the future will depend on my medicine” was the lowest reported in this study. In terms of the concerns, the item “this medicine gives me unfavorable side effects” was predominant, while “having to take medicine worries me” was the lowest apprehension.

A stepwise binary logistical regression model was conducted to describe the associated factors of adherence in the studied sample. A strong prediction of adherence to medications was attributed to the BMQ necessity score (positive correlation), while a negative correlation to adherence was strappingly predicted by the BMQ concern score and frequency of taking medications. Interestingly, having medical insurance also had a negative correlation with adherence (*p* < 0.01). On the other hand, no correlation was found between adherence and gender, educational level, income level and disease duration. These findings are consistent with previous reports that correlated adherence to beliefs about medications. Generally, there as a typical correlation between adherence to medications and beliefs about medications. It is agreed in many reports that necessity beliefs are positive associated factors of adherence behavior, while concern views predicted low rates of adherence [12,20]. Adherence to medications was also found to be reversibly correlated to the daily dosage frequency [21]. Furthermore, once-daily regimens were found to improve the adherence to medications in patients with chronic diseases [22]. An interesting finding in the current study is that insured patients were predicted to have lower adherence rates compared to noninsured patients. It has been reported that medical insurance plays a crucial role in improving the adherence to medications in patients with chronic diseases [23,24]. However, adherence to medications was reported to be negatively affected by insurance type, insurance copayment, drug coverage and prescription cap [25,26]. Similar findings were reported in previous studies conducted to assess the association between beliefs about medications and adherence to medications. Globally, it has been confirmed that there is a statistically significant relationship between medication beliefs and adherence to medications in general chronic diseases patients. The conducted studies portrayed findings and assimilations non-inclusively in countries like Nigeria [27], Germany [28] and China [29] illustrated similar findings. An attitudinal analysis study in elderly patients living alone in South Korea showed that the adherence rate was higher in the accepting group. The other three groups showed significantly lower adherence rates [20]. A study in Swedish patients with stroke revealed that nonadherent patients showed less common positive beliefs and more common negative beliefs toward their medications [30]. Furthermore, similar results were also reported when evaluating the association between beliefs about medications and adherence in specific populations, including diabetes mellitus [31], hypertension [32] and dyslipidemia [33].

Gender, educational level, income level, age and disease duration were found to be not significantly associated with adherence to medications. Previous studies showed, to some extent, inconsistent and opposing findings of these aspects. For example, two studies that investigated the effect of patient’s gender to adherence illustrated that males were more adherent to their chronic medications compared to females [34,35]. In terms of the effect of education level on adherence, lower educational attainment in African Americans was related to higher adherence among men but lower adherence among women [36]. Another study concluded that patient adherence was positively affected by older age and a higher educational level of patients [37]. A statistically significant association was found between the income level and adherence to antidepressants in patients with depression [38]. In another study, adherence increased as age increased until age 69 and started to decrease from age 70 in patients with hypertension [39], and another report that associated between the duration of a treatment and adherence to medication in patients with schizophrenia revealed that there are critical periods during which patients become most predisposed to poor adherence [40]. It could be concluded that the associated factors of adherence can vary widely depending on the study design, disease groups, geographic areas and other factors.

Adherence to medications is widely studied in the literature, as it is a detrimental factor in reducing the mortality and morbidity in chronic diseases and, also, in reducing hospitalization total healthcare costs. A review study [41] showed 19 studies about medication adherence. The rate of medication nonadherence in Middle Eastern countries ranged from 1.4% to 88%. In our study, the adherence rate was calculated to be 32%.

In terms of expressing the necessity and concerns toward their medications, patients attributed the highest and lowest necessities to the statements “My health, at present, depends on my medicine” and “My health in the future will depend on my medicine”, respectively, while the highest and lowest concerns scores were referred to the statements “This medicine gives me unfavorable side effects” and “Having to take medicine worries me”, respectively.

Since 61.1% of the patients had high fears and concern scores about the long-term side effects of taking medications chronically, it is mandatory for pharmacists to be knowledgeable and fully aware of such fears. Pharmacists should also direct patients’ education and intervene to minimize such concerns and, consequently, minimize nonadherence. It is also of immense importance that pharmacists play their role in patient education, as this is well-known to improve the possible beliefs about medications in general and, consequently, contributes to improve adherence to medications. For example, patients who take medications for chronic illness such as hypertension or diabetes mellitus need to know that their medications are not addictive and that the medications have an acceptable safety profile for long-term use. Therefore, the assessment of medication beliefs may be important for the success of medication improvement strategies.

Patients’ concerns lead to hesitancy in taking medication; thus, patients’ education as a core of intervention is recommended to minimize patient myths about their treatment plans. Therefore, addressing the causes behind nonadherence in this study can aid in improving the communication between patients and healthcare providers. Accordingly, the healthcare team can develop treatment strategies according to patients’ hopes and expectations and allow patients to make decisions on therapeutic choices. This conclusion imposes the concept of “concordance to medications “rather than “adherence to medications”. The former concept implies that both physician and patient discuss the treatment plan and the patient indirectly “promises” to adhere to such a discussed plan.

In terms of specific beliefs about medications, patients expressed high levels of concerns about the medications they used (mean score 3.28). This confirms the earlier discussed finding that patients in general are afraid of using their medications.

The results of our altitudinal analysis were consistent with previous research to a good extent [3,21]. Furthermore, the altitudinal analysis results should emphasize the previously mentioned fact that patient education is a key point in pharmacy practices in order to assure the patients to use their medications in the “right way” to get the maximal clinical benefits. In turn, once patients receive adequate “education” about their medications, mainly from the pharmacist, this shall configure positive beliefs about medications and, hence, patients adhere more to their treatment regimens.

Stepwise multinomial logistical regression modeling is a strong statistical tool that is widely used to predict variables. To our knowledge, this is the first study that utilized a logistical regression model to investigate the associated factors of nonadherence in patients with chronic diseases using the BMQ and MARS-5 questionnaires in the study population. The association between adherence to medications and insurance status has not been investigated thoroughly in the studied population’s geographic area. The current study associated insurance status to adherence and, indirectly, to beliefs about medications. Further investigation is urged to explain the association in a more systematic fashion. Furthermore, this is the first time for utilizing the MARS-5 questionnaire as a subjective approach to assess the adherence status in the Jordan adult population.

The median frequency of daily taken medications in the current study was four medications (doses). The regression model suggested that the daily frequency of taking medications is directly proportional to the adherence rates. This finding is consistent with the findings from many previous studies. A meta-analysis suggested that, across the acute and chronic disease states, reducing the dosage frequency may improve the adherence to therapies among patients [42]. Accordingly, it is imperative to utilize different strategies to decrease the total daily dosage frequency, such as combined and once-daily dosage forms, in patients with chronic diseases.

Insurance providers play a crucial role in medication adherence and individual patient’s wellness tracks. Unlike what is expected, having medical insurance was inversely proportional to the adherence rates in the studied sample. This might be attributed to two scenarios. First, having medical insurance may relieve the tension due to the ability to afford chronic medications. This “relief” feeling may influence the patients’ perceptions about the seriousness of their medical conditions, thus negatively affecting their adherence. Second, insurance providers may restrict access to certain medications in certain conditions. This may leave the healthcare provider with less therapeutic or treatment options, which may negatively affect their adherence to medications. The finding that insurance may negatively affect adherence is very interesting and urges further investigation, as this may increase the financial burden on insurance providers due to nonadherence complications and consequences.

There were some limitations identified throughout this study. The data collection in this study was conducted via self-reporting, which may lead to social desirability and recall biases. Furthermore, an interviewer bias may be present in the collected data, despite the fact that the interviewer was well-trained to administer the questionnaires in the same manners for all the respondents without any further explanations. During the data collection, some important variables were possibly not collected in order not to balance between the feasible length of the questionnaire and the amount of data obtained. For example, the Medication Regimen Complexity Index (MRCI), living conditions and severity of the diseases could be potentially associated with adherence, but related data could not be collected.

## Figures and Tables

**Table 1:** Demographic characteristics of the participants (adhered, non-adhered and total).

Sample Characteristics	Adhered Frequency (%) or Mean (SD)	Non-Adhered Frequency (%) or Mean (SD)	Total Frequency (%) or Mean (SD)
**Gender**			
Male	80 (32.5%)	166 (76.5%)	246 (50.7%)
Female	75 (21.4%)	164 (68.6%)	239 (49.3 %)
**Age**	57.34 (13.95)	57.05 (12.27)	57.14 (12.81)
**Educational level**			
Illiterate ^a^	8 (50.0%)	8 (50.0%)	16 (3.3%)
Primary Education	11 (20.0%)	44 (80.0%)	55 (11.3%)
Secondary Education	54 (41.5%)	76 (58.5%)	49 (10.1%)
High School	76 (58.5%)	54 (41.5%)	130 (26.8%)
Bachelor’s Degree	59 (30.1%)	137 (69.9%)	196 (40.4%)
Master’s Degree	9 (33.3%)	18 (66.7%)	27 (5.6)
PhD	3 (75%)	9 (75%)	12 (2.5)
**Income level**			
Low ^b^	16 (29.6%)	38 (70.4%)	57 (11.8%)
Intermediate ^c^	115 (30.7%)	259(69.3%)	374 (77.1%)
High ^d^	24 (42.1%)	33 (57.9)	54 (11.1%)
Diseases			
Hypertension	58 (33.5%)	115 (66.5%)	173 (35.7%)
Diabetes	46 (29.5%)	110 (70.5%)	156 (32.2%)
Cardiovascular	29 (33.0%)	59 (67.0%)	88 (18.1%)
Asthma	17 (32.7%)	35 (67.3%)	52 (10.7%)
**Daily frequency of taking medications**	3.00 (1.35)	3.78 (2.02)	3.53(1.87)
**Ensured**			
Yes	67 (22.3%)	234 (77.7%)	301 (62.1%)
No	88 (47.8%)	96 (52.2%)	184 (87.9%)

^a^: A person who cannot write or read the local language, ^b^: < 1005 USD per anum, ^c^: 1006−12,325 USD per anum and ^d^: >12,325 USD per anum.

**Table 2:** Participants’ responses to the MARS-5 questionnaire.

Medication Adherence Report Scale		Frequency (%)	Mean (SD)
I forget to take them	Always	18 (3.7)	3.21 (1.10)
	Often	116 (23.9)	
	Sometimes	180 (37.1)	
	Rarely	87 (17.9)	
	Never	84 (17.3)	
I change the dose	Always	8 (1.6)	3.46 (1.10)
	Often	91 (18.8)	
	Sometimes	176 (36.3)	
	Rarely	91 (18.8)	
	Never	119 (24.5)	
I stop taking them for a while	Always	13 (2.7)	3.39 (1.13)
	Often	93 (19.2)	
	Sometimes	194 (40)	
	Rarely	63 (13)	
	Never	122 (25.2)	
I decide to skip a dose	Always	6 (1.2)	3.51 (1.12)
	Often	89 (18.4)	
	Sometimes	178 (36.7)	
	Rarely	78 (16.1)	
	Never	134 (27.6)	
I take medications less than instructed	Always	15 (3.1)	3.49 (1.14)
	Often	77 (15.9)	
	Sometimes	182 (37.5)	
	Rarely	77 (15.9)	
	Never	134 (27.6)	

**Table 3:** Responses to the BMQ items (specific necessity and specific concerns).

Statement	Agree/Strongly Agree N (%)	Mean (SD)
**Specific necessity**		3.67 (0.71)
1—My health, at present, depends on my medicine	417 (86)	4.05 (0.94)
2—My life would be impossible without my medicine	406 (83.7)	3.75 (0.86)
3—Without my medicine, I would be very sick	296 (61)	3.58 (0.89)
4—My health in the future will depend on my medicine	199 (41)	3.33 (0.76)
5—My medicine protects me from becoming wore	275 (59.7)	3.62 (0.84)
**Specific concerns**		3.28 (0.84)
1—Having to take medicine worries me	137 (28.2)	3.02 (0.90)
2—I sometimes worry about the long-term effects of my medicine	296 (61.1)	3.37 (0.98)
3—My medicines are a mystery to me	246 (50.7)	3.24 (0.95)
4—My medicine disrupts my life	264 (54.4)	3.13 (1.10)
5—I sometimes worry about becoming too dependent on my medicine	229 (47.2)	3.32 (0.90)
6—This medicine gives me unfavorable side effects	313 (64.5)	3.59 (0.97)

**Table 4:** Stepwise binary logistical regression of the variables associated with adherence.

Significant Variables *	B	*p*-Value	Odds Ratio	Confidence Interval of 95%	
				Lower	Upper
**Necessity score**	1.44	<0.01	4.22	2.59	6.87
**Concerns score**	−0.32	0.02	0.73	0.56	0.95
**Frequency of taking medications**	−0.30	<0.01	0.74	0.65	0.84
**Medical insurance**					
Ensured	−1.37	<0.01	0.26	0.16	0.40
Not ensured	Reference	-	-	-	-

*: Stepwise binary logistical regression; variables included in the model: gender, educational level, income level, medical insurance, necessity score, concerns score, frequency of taking medications, age and disease duration.

## Data Availability

The data presented in this study are openly available in Zenodo data repository at 10.5281/zenodo.5501405.
